# A Sheet-Shaped Transforming Robot That Can Be Thrown from the Air

**DOI:** 10.3390/biomimetics9050287

**Published:** 2024-05-11

**Authors:** Naoki Iida, Mitsuharu Matsumoto

**Affiliations:** Graduate School of Informatics and Engineering, University of Electro-Communications, 1-5-1, Chofugaoka, Chofu-shi, Tokyo 182-8585, Japan; i2230011@edu.cc.uec.ac.jp

**Keywords:** transforming robot, sheet-shaped robot, throwable robot, system development

## Abstract

In this paper, we describe a sheet-shaped throwable transforming robot. Sheet-type robots can change their shape to perform tasks according to the situation. Therefore, they are expected to be useful in places with many restrictions, such as disaster sites. However, most of them can only move slowly on the ground. Therefore, in order to actually deliver the robot to the disaster site, it must be carried manually. To solve this problem, we are developing a sheet-shaped robot that can be thrown from the sky. Previously developed prototypes could only move in the forward direction, and the transition from falling to walking was complicated and uncertain. In this paper, we report on a new prototype that improves on these shortcomings.

## 1. Introduction

Various studies on transforming robots have been reported so far. Several studies have reported transformable robots that can change the way they move depending on the situation. As an example, Kosett et al. proposed a transforming robot with a wheel mode and a helicopter mode [1]. Boria et al. also proposed a robot that can fly and walk [2]. Modular self-reconfiguring robots (MSRs) are an alternative approach to transforming robots to achieve robots that adapt to changes in their surrounding environment. According to a study on MSRs by Yim et al. [3], the first research on MSRs was reported in the 1980s [4]. Many researchers are conducting research on MSRs since then. Examples include the Pollybot proposed by Yim et al. [5], the Crystalline system proposed by Gilpin et al. [6], and M-TRAN proposed by Murata et al. [7,8,9].

Many MSR transformations are achieved by mechanically changing the module-to-module connections. To achieve this connection, the module structures in MSRs tend to be relatively complex and large in size. When connecting modules, it is necessary to identify each other’s positions.

Sheet-type robots are being researched as another approach to deformable robots. A sheet-type robot is a robot that can create various three-dimensional shapes by bending its two-dimensional body. Sheet-type robots have the advantage of being able to form a three-dimensional shape from a two-dimensional shape, and can occupy a very small volume. Several studies on robots that create three-dimensional structures from two-dimensional structures have been reported so far. For example, programmable matter is a deformable device proposed by Hawkes et al. [10,11]. They used magnets and shape memory alloys to achieve the transformation from a flat surface to various shapes. Related work can also be found in the field of self-organization. For example, Boncheva et al. used magnetic force to create a three-dimensional closed circuit from a sheet [12].

Additionally, many studies on disaster response robots have been reported in recent years. These robots are being developed to rescue victims in the event of a flood or earthquake [13,14,15,16,17]. In the event of a disaster, it is extremely important to carry out exploration activities quickly. Disaster exploration using such robots includes aerial exploration approaches and land exploration approaches.

Unmanned aerial vehicles (UAVs) are a technology that is expected to be used for aerial searches during disasters. Many UAVs have been proposed due to their potential [18,19,20,21,22,23,24,25]. However, many UAVs operate on batteries, which has the disadvantage of short flight times. UAVs may be difficult to use depending on weather conditions. Ground-based exploration robots offer more stable exploration than aerial exploration. However, in order to realize ground-based exploration, it is necessary to transport the robot to the disaster site.

In order to solve the problems above, we are working on developing a disaster relief robot that can be thrown from the air. The envisioned system would first use unmanned aerial vehicles to disperse large numbers of robots over the disaster area. The distributed robots next move on the ground and carry out exploration activities.

We look to sheet-type robots to achieve this goal. Sheet-type robots have a thin sheet-like structure that can be deformed into various shapes. Therefore, it may be possible to achieve both adaptability to disaster-stricken areas and miniaturization and cost reduction [26,27].

Most disaster robots are heavy and must be carried manually to the disaster site because they emphasize work efficiency at the disaster site. In contrast, sheet-type robots are lightweight and flexible. If such robots can be thrown from the sky safely, they can be installed at disaster sites quickly. To this end, previous research reported on a sheet-shaped robot that mimics plant seeds and can be thrown from the air [28]. However, the developed robot could only move forward, and the transition from falling to walking was complicated and uncertain. Therefore, in this paper, we introduce a new robot that simplifies the transformation from a falling mode to a walking mode and achieves forward, backward, left, and right movements.

In Section 2, we perform a theoretical analysis of the robot design and identify parameters that reduce the falling speed in the next section. In this research, we aim to reduce the falling speed of robots by considering the design of robots that imitate plant fruits. In order to clarify what kind of plant should be imitated, we briefly describe our past research [28]. We also formulate the most promising physical model for a dipterocarp based on our past results [28]. Section 3 reports on the results of falling and walking experiments on the developed robot. In the falling experiment, we experimentally investigated the parameters that would best reduce the robot’s falling speed. In the walking experiment, we confirmed that the robot could move in four directions, i.e., forward, backward, left, and right, and quantitatively confirmed its walking speed. Conclusion and future prospects are given in Section 4.

## 2. Theoretical Analysis

### 2.1. Fall Prevention Effect of Plant Fruit Wings

Bio-inspired technologies range from software to hardware [29]. Many biomimetic robots have also been proposed for robot movement from the air to land [30]. For a robot that imitates animals, some studies have mimicked the pitch control mechanisms of reptiles [31]. Other authors have created a robot that mimics the aerial stability of squirrels [32].

Alternatively, there are also robots that imitate plants. Unlike many animals, flight in plants is passive [33,34]. The envisioned robot would be difficult to control in a complex manner, and it would be desirable to have a simple structure that would reduce the impact when it falls from the air. Therefore, in this study, we investigated the structure of a robot that imitates the structure of plants.

In this research, we use the wing fruits of plants as a reference for the method of throwing robots from the air. Some plants use the power of the wind to disperse their seeds. Seeds that are dispersed by the wind have a shape that enables them to easily fly in the air. Seeds that leave the tree slow down in the air and travel with the wind. The structure of these seeds can be imitated using origami, making it possible to apply them to sheet-type robots.

Plant seeds do not use strings, unlike the parachute method, and their structure slows down their fall. Hence, it is expected that they will be able to fall without becoming entangled with each other even if there are a large number of robots. Additionally, the robot will reduce its speed when falling, even if users do not control the robot in the air.

**Figure 1:**
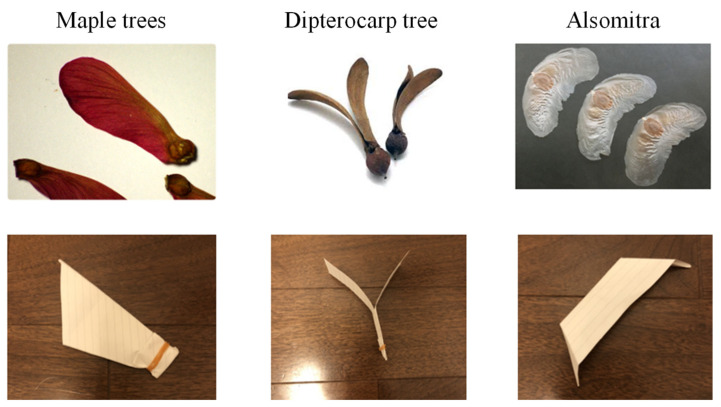
Examples of wing fruit.

**Figure 2:**
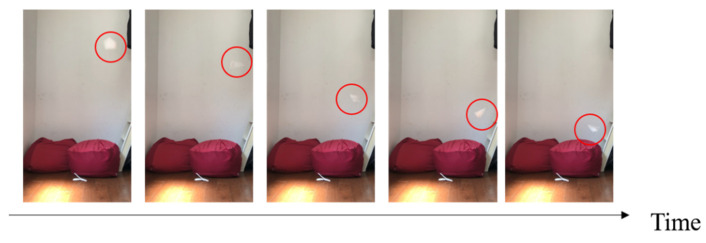
Drop test of the maple tree model. The red circle is shown to clarify the location of the falling model.

**Figure 3:**
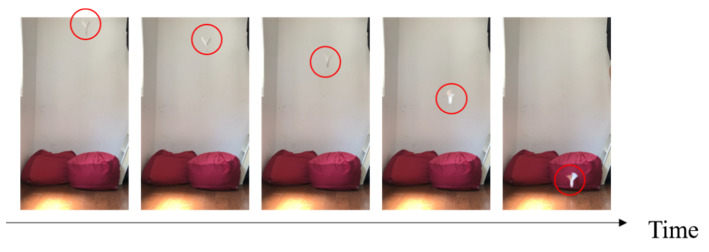
Drop test of the dipterocarp model. The red circle is shown to clarify the location of the falling model.

**Figure 4:**
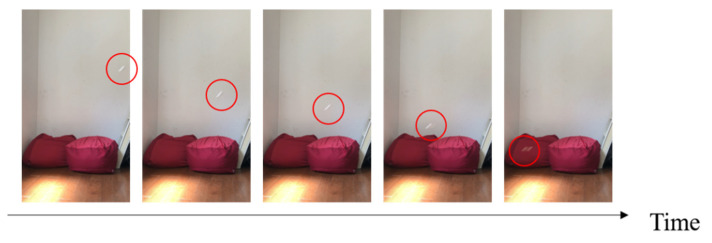
Drop test of the alsomitra model. The red circle is shown to clarify the location of the falling model.

Three candidates were selected as robot models: a maple tree, a dipterocarp tree, and an alsomitra tree [28]. Figure 1 shows the model plant and its origami imitation. Figure 2, Figure 3 and Figure 4 show the drop test of the maple tree model, the dipterocarp tree model, and the alsomitra model, respectively. To improve visibility, falling models are circled in red. The maple model has a simple structure, but depending on how it is dropped, it may fall without rotating. The alsomitra model resembles a paper airplane and glides down, making it difficult to land at a target location from above. In contrast, the dipterocarp model easily rotates and remains stable when dropped. Based on these experiments, we selected the dipterocarp model as the robot’s falling model.

### 2.2. Four-Blade Origami Robot Spinning and Falling

**Figure 5:**
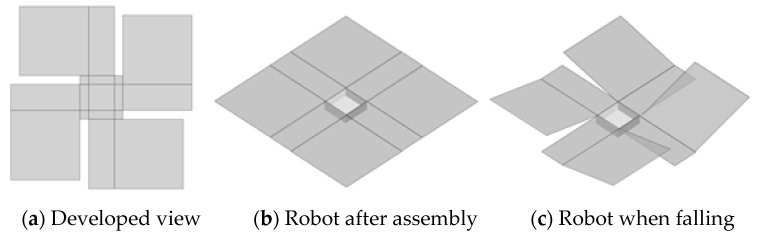
Overview of developed sheet-shaped robot.

In this section, we consider the physical model of the robot and investigate the factors that determine its rotation and its speed during a fall. Figure 5 shows the overview of the developed sheet-type robot. The sheet-type robot consists of four wings. Considering future developments, the shape was designed to be close to a rectangle, allowing for more diverse folding methods.

### 2.3. Energy Conservation Law

We can express the law of conservation of energy when the robot falls from the height *H* to the height *h* while it is rotating as follows:

(1) $$MgH=\frac{1}{2}MV^{2}+\frac{1}{2}I\omega^{2}+Mgh,$$

where *M* represents the model mass. *V* represents the falling velocity. *I* represents the moment of inertia of the wing. ω represents the angular velocity of the rotating wing. By transforming Equation (1), *V* can be written as follows.

(2) $$V=\sqrt{\;2g\left(H-h\right)-\frac{I\omega^{2}}{M}}$$

From Equation (2), *V* increases as the moment of inertia *I* and angular velocity ω decrease. As mass *M* decreases, *V* decreases.

The general explanation of the moment of inertia is as follows. Let us consider N objects. *m_i_* represents the mass of the *i*th object. The moment of inertia *I* can be written as follows using mass *m_i_* at distance *r_i_* from the rotation axis.

(3) $$I=\sum_{i}m_{i}r_{i}$$

Therefore, as the mass of the part of the blade that is farther from the axis of rotation increases, the moment of inertia increases.

### 2.4. Rotation Mechanism

**Figure 6:**
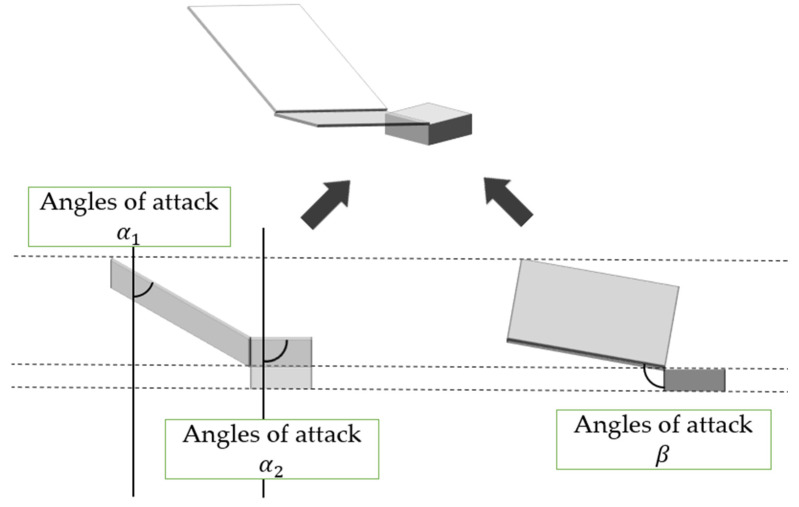
Relation between the robot wing and the angle of attack.

The angle of attack in the proposed robot is shown in Figure 6. Three angles of attack α_1_, α_2_, and β can be defined for a wing of mass *m* as shown in Figure 6. When the robot falls, air pushes up its wings. Therefore, a drag force *d* and lift forces *l*_α_ and *l*_β_ (0 < α_1_, α_2_ < π/2 < β < π) are generated.

**Figure 7:**
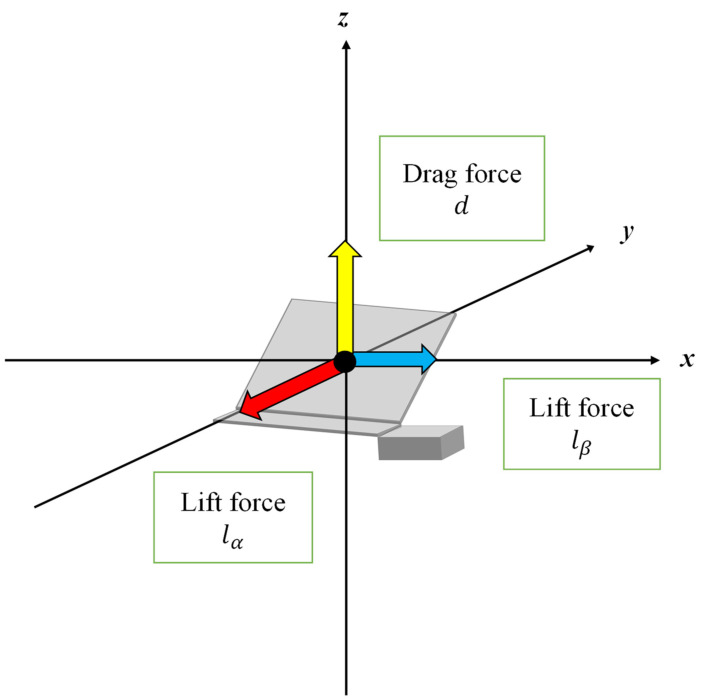
The force that acts on a robot when it falls from the air.

Figure 7 depicts the force that acts on a robot when it falls from the air. In Figure 7, we defined three forces: *d*—the drag force, *l_a_*—the lift force due to *a*, *l_b_*—the lift force due to *b*. Here, the drag force is defined as a force that acts on an object moving in a fluid, and has the same direction parallel to the velocity of the flow in fluid dynamics. In this case, the robot is in free fall, and the fluid flows vertically upwards towards the robot. Hence, the direction of d is vertically upward. On the other hand, the lift force is defined as the force exerted on an object moving in a fluid perpendicular to the direction of movement of the object fluid dynamics. The lift generated here consists of a force derived from a and a force derived from b, but both are horizontal to the ground.

**Figure 8:**
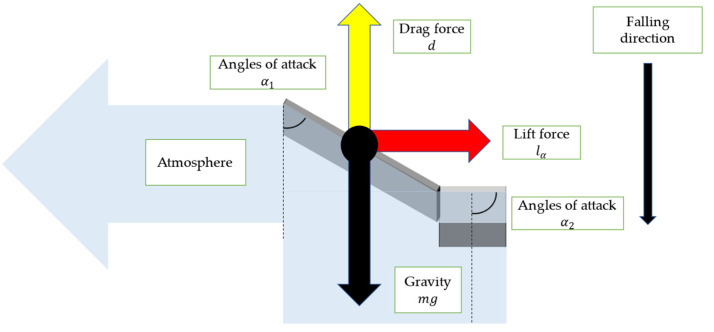
Cross-section of wing, and lift force *l*_α_ with angle of attack α_1_ and α_2_.

Figure 8 is a cross-section view of the robot wing when viewed from the direction of α_1_ and α_2_ in Figure 6. It shows the angles of attack α_1_ and α_2_ and lift force depending on α_1_ and α_2_.

**Figure 9:**
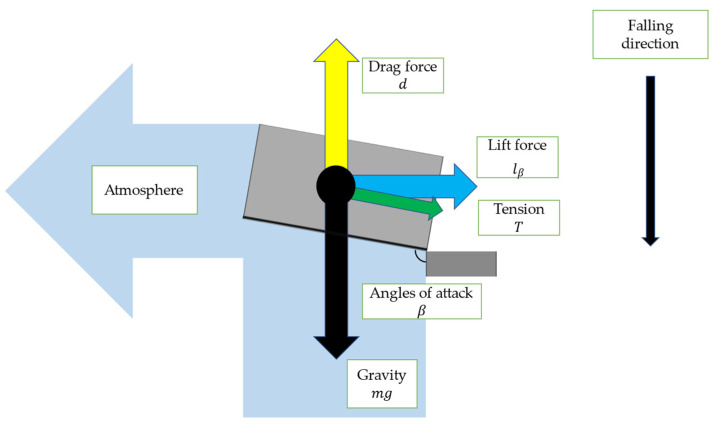
Lift force *l_β_* due to angle of attack β.

Figure 9 is a cross-section view of the robot wing when viewed from the direction of β in Figure 6. It shows the angle of attack β and lift force depending on β.

When the air pushes against the robot’s wings, it creates a lift force *l*_α_ tangential to the circular motion, causing the model to rotate. The lift force *l*_β_ acts as a centripetal force for the rotation of the robot.

### 2.5. Drag and Lift Forces Acting on the Robot

In fluid dynamics, the drag force can be described using the drag equation. The equation is attributed to Lord Rayleigh [35]. We can express the drag force *d* is as follows:

(4) $$d=\;\frac{C_{d}S\rho V^{2}}{2}$$

where *C_d_* represents the drag coefficient. *S* is the wing area as the reference area. *ρ* represents the density of the atmosphere. *V* is the relative velocity of the object and fluid. When we assume that the atmosphere is stationary, the falling velocity *V* is the corresponding velocity.

**Figure 10:**
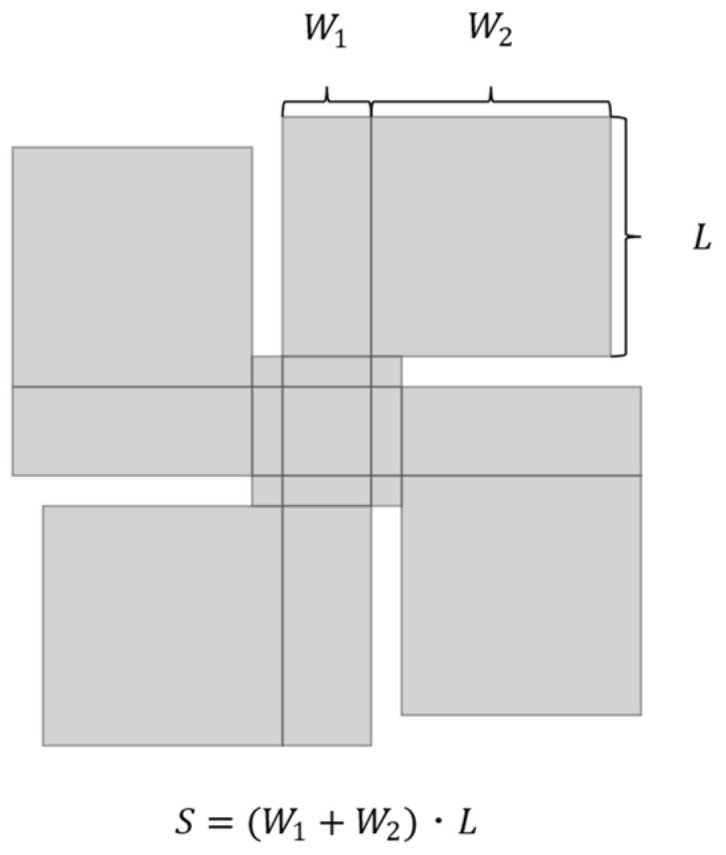
Representative area.

We can express the lift forces *l_α_* and *l_β_* due to α and β as follows:

(5) $$l_{\alpha }=\;\frac{C_{l_{\alpha }}S\rho V^{2}}{2}$$

(6) $$l_{\beta }=\;\frac{C_{l_{\beta }}S\rho V^{2}}{2}$$

where $C_{l_{\alpha }}$ and $C_{l_{\beta }}$ indicate the coefficients of the lift forces *l_α_* and *l_β_*, respectively. As shown in Figure 10, the representative area *S* can be expressed as follows:

(7) $$S=\left(W_{1}+W_{2}\right)L$$

where *L* represents the length of the wing. *W*_1_ and *W*_2_ are the widths defined in Figure 10. Hence, we can describe *d* as:

(8) $$d=\;\frac{C_{d}\left(W_{1}+W_{2}\right)L\rho V^{2}}{2}$$

We also describe *l_α_* and *l*_β_ as follows:

(9) $$l_{\alpha }=\;\frac{C_{l_{\alpha }}\left(W_{1}+W_{2}\right)L\rho V^{2}}{2}$$

(10) $$l_{\beta }=\;\frac{C_{l_{\beta }}\left(W_{1}+W_{2}\right)L\rho V^{2}}{2}$$

In fluid dynamics, the angles of attack are defined as the angle between a reference line on a body and the air flow vector. A typical aircraft travels horizontally, so the velocity of the fluid is parallel to the ground. In contrast, the robot we created falls vertically, so the velocity of the fluid is perpendicular to the ground. Therefore, it is considered that lift force acts horizontally to the ground and drag force acts perpendicularly to the ground.

Because the direction of lift and drag is different from that of a normal aircraft, the effect of lift and drag on falling is also different from that of a normal aircraft. In a normal aircraft, the force that acts vertically upward is lift force, and lift force maintains the aircraft’s attitude in the air. In contrast, in the developed robot, the force that acts vertically upward is a drag force. Additionally, the force directly reduces the speed at which it falls to the ground. Lift force acts as a force that rotates the robot. This rotation also attenuates the robot’s falling speed. The drag force on an object is determined by the density of the fluid, its relative velocity with the fluid, and the reference area as described in Equation (4). Among these parameters, the reference area changes depending on the angles of attack.

It is known that the lift of an aircraft wing increases until the angle of attack increases to a certain extent, but once the angle exceeds a certain point, the aircraft stalls. In the developed robot, the lift force acts as a force that rotates the robot. Hence, it is thought that the rotation force becomes large up to a certain angle.

Alternatively, drag force is a force that acts vertically upwards on the robot and directly impedes the robot’s falling speed. When the angles α and β change, the projected area of the blade as seen from the flow direction changes. As α becomes larger, the projected area becomes larger. As a result, the surface area of the wing that receives the fluid increases, increasing drag force. On the other hand, as β becomes larger, the projected area becomes smaller. As a result, the surface area of the wing that receives fluid is reduced, reducing the drag force.

In our formula, we include the influence of α and β in the drag coefficient.

The drag coefficient *C_d_* is a dimensionless coefficient. *C_d_* increases as the angles of attack *α*_1_ and *α*_2_ increase, and decreases as the angle of attack β increases (0 < *α*_1_, *α*_2_ < π/2 < *β* < π). Dynamic pressure is defined by the kinetic energy of a fluid per unit volume.

The lift coefficient $C_{l_{\alpha }}$ is also defined as a dimensionless coefficient. As long as *α*_1_ and *α*_2_ are small, it increases as *α*_1_ and *α*_2_ increase. However, when α_1_ and α_2_ exceed a certain angle, it decreases (0 < *α*_1_, *α*_2_ < *π*/2). It also decreases as the angle of attack *β* increases (*π*/2 < *β* < *π*).

The lift coefficient $C_{l_{\beta }}$ has similar properties to $C_{l_{\alpha }}$. However, the properties for *α*_1_, *α*_2_, and β are reversed. As long as *β* is small, it increases as *β* increases. However, when *β* exceeds a certain angle, it decreases (*π*/2 < *β* < *π*). It also decreases as the angles of attack α_1_ and α_2_ increase (*π/2 <* α_1_, α_2_ < *π*).

### 2.6. Equation of Motion

**Figure 11:**
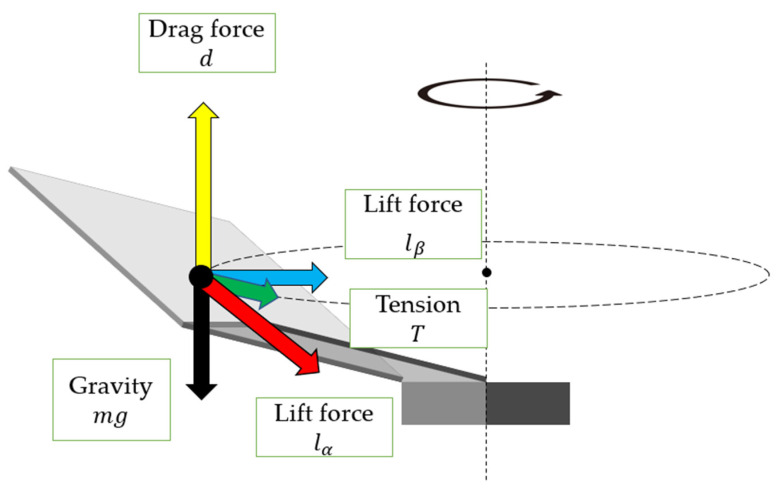
Rotational motion of a single wing.

Figure 11 shows the relationship between the force acting on one of the robot wings and the rotation caused by that force. When the robot rotates and falls, β is stable. We can obtain the following equation of balance:

(11) d=mg+Tcos⁡(π−β)

We can obtain the following equation from the equation of motion regarding rotation:

(12) $$m\frac{L}{2}\sin\left(\pi -\beta \right)\frac{d\omega }{dt}=l_{\alpha }$$

(13) $$m\frac{L}{2}\sin\left(\pi -\beta \right)\omega^{2}=l_{\beta }+T\sin\left(\pi -\beta \right)$$

Here, Equation (12) represents an equation of motion for the tangential direction of rotational motion. Equation (13) represents an equation of motion with respect to the center direction of rotational motion. If we integrate both sides of Equation (12) by *t* and rearrange it, we can transform it into the following equation:

(14) $$\omega =\frac{C_{l_{\alpha }}\;\left(W_{1}+W_{2}\right)\;\rho V^{2}t}{m\sin\beta }$$

When the rotational motion is uniform circular motion, ω can be expressed as follows using Equation (13) and using Equations (8), (10), and (11):

(15) $$\omega =\sqrt{\frac{2\left(C_{l_{\beta }}\cos\beta -C_{d}\;\sin\beta \right)\left(W_{1}+W_{2}\right)\rho V^{2}}{m\sin2\beta }+\frac{2g}{L\cos\beta }}$$

Furthermore, when the rotational motion is a uniform circular motion, the values of Equations (14) and (15) are equal.

We fixed β to π since the friction surface during walking becomes unstable when the angle of attack β is changed. α_2_ was also fixed since α_2_ is a movable part during walking and it is desirable not to move it. Using α_1_ as a variable, we experimentally investigated how much the falling speed is reduced depending on the value of α_1_.

## 3. Experiments

### 3.1. Configuration of Sheet-Type Robot

In this section, we describe the concrete configuration of the developed sheet-type robot. The main components are listed as follows: polypropylene (PP) board, biometal (BMX), natural rubber, steel, and copper foil round terminals (oxygen-free copper)

PP sheets are used for the body of the sheet-type robot, which is the same as for the previous prototype. PP sheets were used because they are easy to deform and have the lowest density of all plastics at 0.9 to 0.91, making them suitable for being thrown from the air. The melting point of PP is 100 °C to 140 °C, which is relatively high among general-purpose plastics. However, when BMX is heated, it may exceed the melting point of PP. Hence, there is a concern that it may melt depending on the conditions. To prevent the PP from melting due to the heat generated when driving the BMX, the contact area between the PP board and the BMX was reduced using round terminals.

**Figure 12:**
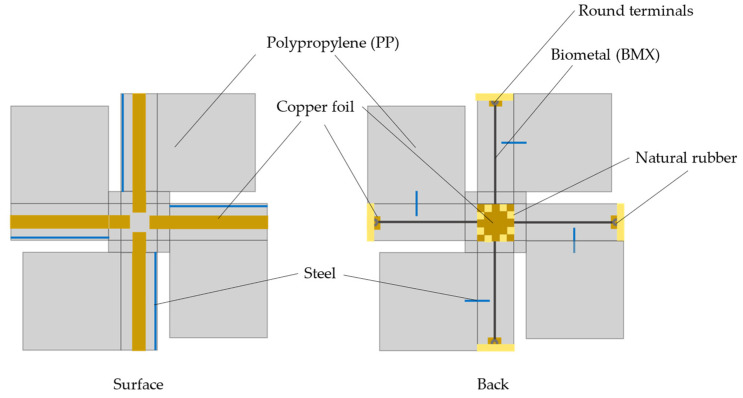
Components of the sheet-shaped robot.

The use of copper foil as a circuit minimizes the effect of conductors on walking. The wings are reinforced with steel wires to prevent distortion of the wings during walking. The ground surface is covered with natural rubber to increase friction. Figure 12 shows the actual configuration of the sheet-shaped robot.

**Figure 13:**
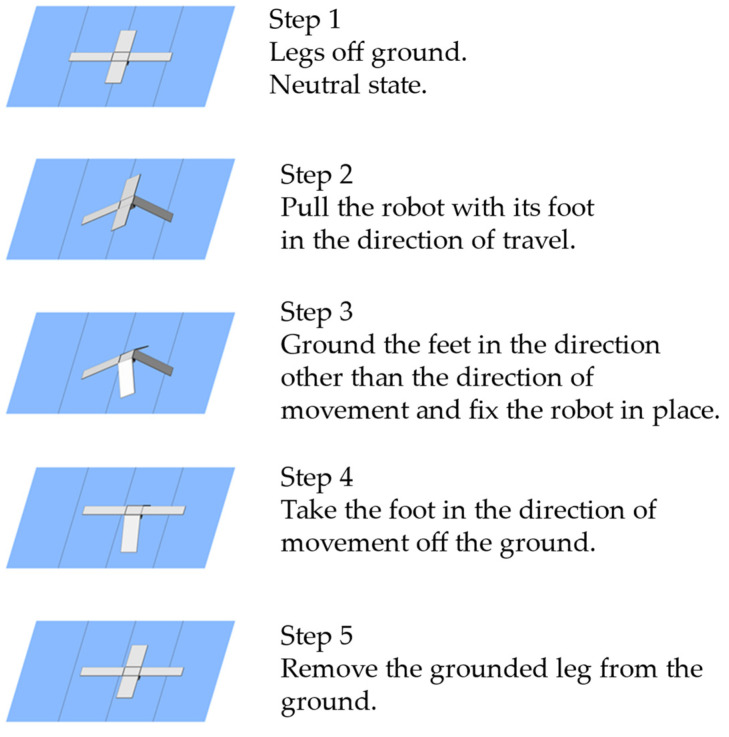
Walking mechanism of the sheet-shaped robot with simplified wings.

Figure 13 shows the walking mechanism of the proposed robot. The robot movement is realized according to the procedure shown in Figure 13. In Steps 1 and 2, the robot drags the body with its forward foot in the direction of travel. If the front foot is returned to its original position, the body also returns to its original position. Therefore, in Step 3, the other feet are grounded to fix the position of the fuselage. In Step 4, the forward leg returns to its original position. In Step 5, the grounded leg is lifted off the ground.

These five steps are repeated to move the robot forward, backward, left, or right.

**Figure 14:**
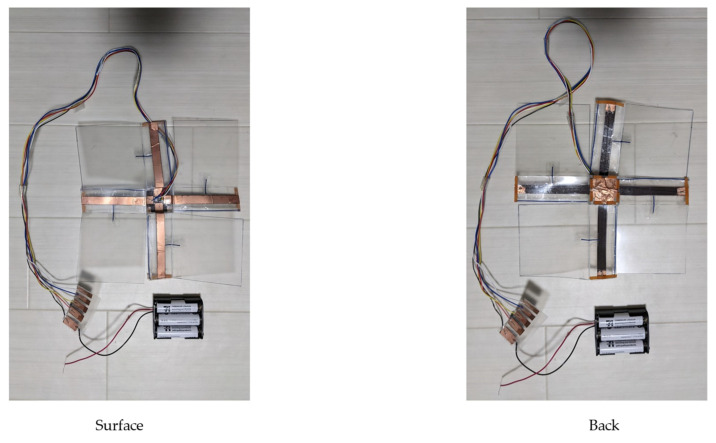
Overview of the developed robot.

Figure 14 shows the overview of the developed robot. The robot is made of PP board with one fold and slit. The body is a rectangular body with a length of 3 cm, a width of 3 cm, and a height of 1 cm. The wings are 8 cm long and 11 cm wide. The mass of the body alone is approximately 11.5 g. Power during operation was supplied externally. The movement of the BMX was controlled manually.

### 3.2. Walking and Falling Experiments

**Figure 15:**
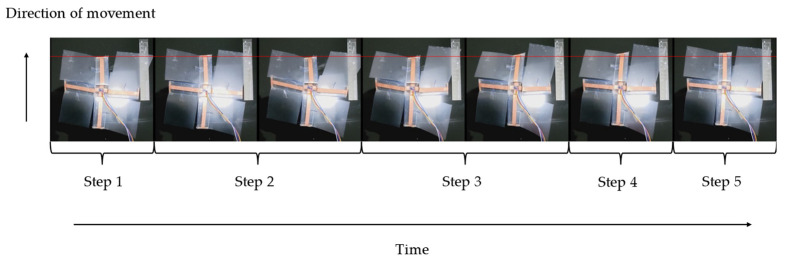
Walking process of the developed robot.

**Figure 16:**
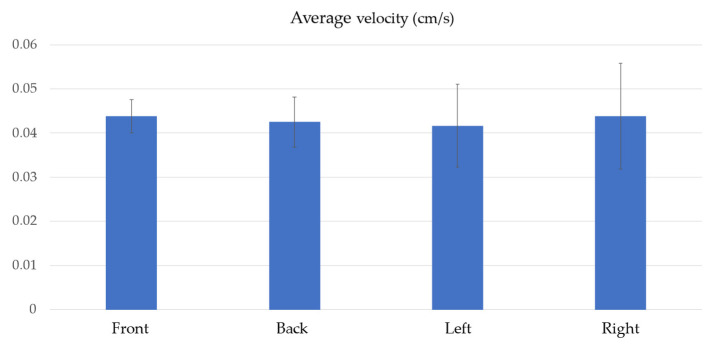
Results of the walking experiment.

The falling speed and moving speed of the sheet-shaped robot were measured using video analysis, and the robot’s walking ability and fall prevention performance were evaluated. Figure 15 shows the procedure of the robot walking. Figure 16 shows the walking speed depending on the walking direction. The experiment was conducted five times. The movement speed was 0.0439 ± 0.00377 [cm/s] to the front, 0.0425 ± 0.00567 [cm/s] to the back, 0.0417 ± 0.00941 [cm/s] to the left, and 0.0438 ± 0.01202 [cm/s] to the right. The average speed was 0.0442 ± 0.00774 [cm/s].

We also conducted a falling experiment and measured its falling speed depending on *α*_1_.

**Figure 17:**
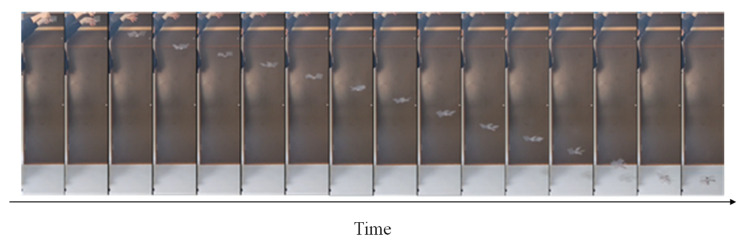
Falling process of the developed robot.

**Figure 18:**
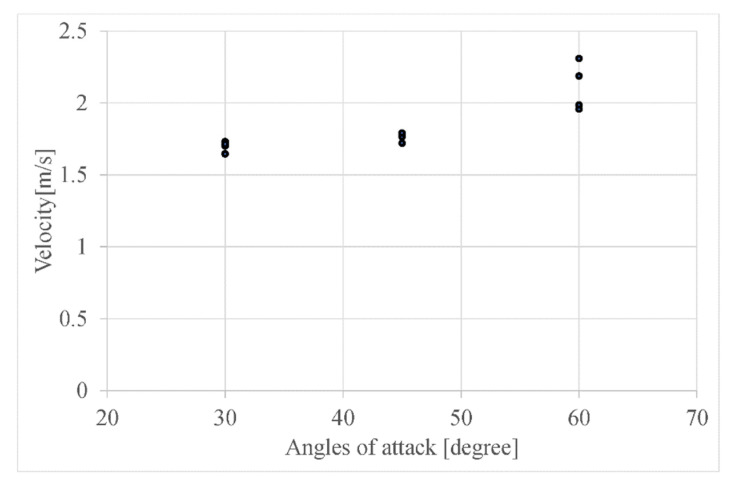
Relation between the angles of attack and the falling velocity.

We varied *α*_1_ from 30 degrees to 60 degrees in 15-degree increments and measured the falling speed and angular velocity when dropped from 2 m. Figure 17 shows the procedure of the robot falling. Table 1 shows the results of the falling experiments. It contains the mean and standard deviation of speed. The lowest falling speed was when *α*_1_ was 30 degrees. Figure 18 shows the relation between the angles of attack and the falling velocity to show the results in details. 

### 3.3. Discussion

Motion experiments confirmed that the sheet-shaped robot is capable of rotating and falling, and moving back and forth, and left and right, on the ground, all in one form. Based on the results of the walking experiments, we discuss the drop, deformation, and movement of the robot.

During falling, the robot succeeded in slowing down its speed by rotating and falling, as in the prototype that imitated a dipterocarp. However, the prototype has more movable parts than the prototype, which may interfere with each other during a mass drop, making it difficult for the prototype to maintain its posture and form during the drop. Furthermore, it still needs to be tested to ensure that it will work properly when subjected to wind pressure from aircraft propellers and strong winds in the sky. In addition, a control system must be mounted on the fuselage to create a self-supporting sheet-shaped robot. To protect the precision machine from the impact of a fall, it must be equipped with a mechanism to further decelerate or soften the impact of a fall.

The robot can move forward, backward, left, and right with its four joints. We used lightweight PP to facilitate the robot’s bending motion using BMX. However, due to the characteristics of the ground crawling method, the robot cannot climb over steps, and its movement speed is not stable depending on the condition of the ground surface. The experiment was conducted on a flat table. Hence it may not work properly if the ground condition is poor. Furthermore, although the average speeds in each direction were close to each other, the speeds had a large standard deviation of 0.0120 [cm/s]. The standard deviation itself also varied in each direction. This may be due to the unstable bending and stretching speeds of each joint due to the elasticity of PP, in addition to the unstable friction surface and the condition of the ground surface as described above. The instability of the movement speed can be improved by adjusting the angle and timing of bending each joint strictly, or by using a structure with anisotropic friction on the friction surface.

Regarding the scalability of deformation, the assembled shape is close to a square, and it may be possible to perform various deformations using a traditional origami folding diagram. This research was limited to ensuring the scalability of the deformation, and further study is needed to determine how to utilize the ineffective sheet-shaped robot in disaster-stricken areas.

## 4. Conclusions and Future Prospects

In this study, we developed a sheet-shaped robot that slows down the fall by rotating after being thrown from the air. The robot can move forward, backward, left, and right after landing.

Conventional disaster response robots require additional parts to expand their functions to adapt to the environment, which increases the volume, weight, and cost of the device, and complicates the control mechanism. We expect that sheet-shaped robots have the potential to solve the problem of size bloat by enabling the robot to adapt to its environment by transforming from a flat surface to various shapes and extending its functionality. However, most of them use deformation only to improve storability, and even those that retain the freedom of deformation have problems in terms of transportation to disaster sites for the purpose of disaster response, due to their slow movement speed.

To solve these issues, we created a sheet-like robot with a simpler structure and greater freedom of movement than past prototypes. We believe that this will solve the problem of the slow movement speed inherent to origami robots in mobilizing them to the disaster site. Since it is desirable for the developed robot to reduce the speed of falling from the sky with a simple structure, we incorporated biomimetic robot technology. By referring to the structure of plants, the speed from the air can be effectively reduced without complicated controls.

The contributions of the developed sheet-shaped robot are summarized as follows: The shape of the designed robot allows it to reduce its falling speed after being thrown into the air. It can move back and forth, and left and right, on the ground after landing, unlike the previous prototype [28]. Since the robot shape is close to a square, we expect to make use of the characteristics of general origami. As the robot is lightweight, thin, and compact, it could be easily stored and transported by the airplane. This study suggests the possibility of aerial dispersal of robots as a new method for deploying disaster response robots and origami robots.

Future challenges include the improvement of the robot control and its movement. As the robot is currently controlled manually, it is necessary to implement a control mechanism using a microcontroller such as an Arduino in order for it to move autonomously. As the developed robot moves slowly and cannot climb hills, it is necessary to improve its movement method. It is also necessary to perform a simulation analysis to examine the relation between the theoretical analysis and the experimental results.

We would like to solve these problems and eventually realize a sheet-shaped robot that can be useful in searching for victims and saving lives, and in evacuation centers.

## Figures and Tables

**Table 1:** Falling velocity for angle of attack *α*_1_.

$\mathrm{Angle}\;\mathrm{of}\;\mathrm{Attack}\;\alpha_{1}$ (Rad)	Falling Velocity (m/s)
2π/12	1.69 ± 0.04
3π/12	1.77 ± 0.03
4π/12	2.08 ± 0.16

## Data Availability

Data are contained within the article.
